# Supplementary material for: Long-term trends in obesity and overweight in women in Ghana from 2003–2023
Source: Commun Med (Lond). 2025 Aug 7;5:338. doi: 10.1038/s43856-025-01082-4 (PMC12332120; doi:10.1038/s43856-025-01082-4)
Supplement: Supplementary file 2 — Supplementary Information [file 43856_2025_1082_MOESM2_ESM.pdf]

Supplementary Material

Table S1: Age-period-cohort regression results: Estimated odds (OR) of obesity compared to healthy weight and 95% confidence interval, Ghana (2003-2022)

Obesity	OR	95% Confidence Interval				OR	95% Confidence Interval		
2-YEAR AGE GROUP					*PERIOD (YEAR)*				
15-17 years old	0.34	0.21	0.57		2003	1	-	-	
18-19 years old	0.53	0.32	0.88		2008	1.37	1.11	1.69	
20-21 years old	1	-	-		2014	2.73	2.05	3.64	
22-23 years old	1.33	0.87	2.02		2022	3.95	2.58	6.04	
24-25 years old	2.08	1.33	3.26						
26-27 years old	3.86	2.50	5.96		*10-YEAR BIRTH COHORT*				
28-29 years old	4.11	2.65	6.36		1953-59	1	-	-	
30-31 years old	5.60	3.60	8.73		1960-69	0.83	0.58	1.19	
32-33 years old	6.37	4.00	10.15		1970-79	0.78	0.47	1.28	
34-35 years old	6.46	3.82	10.92		1980-89	0.64	0.33	1.25	
36-37 years old	7.34	4.29	12.57		1990-99	0.59	0.25	1.38	
38-39 years old	9.32	5.41	16.05		2000-07	0.93	0.32	2.69	
40-41 years old	8.83	5.00	15.57						
42-43 years old	9.25	5.12	16.71						
44-45 years old	6.55	3.34	12.82						
46-47 years old	7.44	3.69	14.98						
48-49 years old	6.88	3.36	14.10						

Note: 14998 individual observations used in the analysis.

Table S2: Age-period-cohort regression results: Estimated odds (OR) of overweight compared to healthy weight and 95% confidence interval, Ghana (2003-2022)

	OR	95% Confidence Interval				OR	95% Confidence Interval		
2-YEAR AGE GROUP					*PERIOD (YEAR)*				
15-17 years old	0.48	0.39	0.60		2003	1	-	-	
18-19 years old	0.78	0.62	0.98		2008	1.34	1.16	1.56	
20-21 years old	1	-	-		2014	1.92	1.56	2.38	
22-23 years old	1.29	1.01	1.66		2022	2.24	1.63	3.08	
24-25 years old	1.71	1.32	2.22						
26-27 years old	1.82	1.39	2.39		*10-YEAR BIRTH COHORT*				
28-29 years old	2.51	1.90	3.30		1953-59	1	-	-	
30-31 years old	2.33	1.74	3.11		1960-69	0.98	0.72	1.33	
32-33 years old	2.77	2.05	3.74		1970-79	0.91	0.61	1.35	
34-35 years old	2.37	1.66	3.37		1980-89	0.91	0.54	1.52	
36-37 years old	2.64	1.82	3.81		1990-99	0.77	0.40	1.48	
38-39 years old	2.60	1.78	3.82		2000-07	0.68	0.31	1.52	
40-41 years old	2.68	1.79	4.01						
42-43 years old	2.46	1.62	3.75						
44-45 years old	2.40	1.48	3.88						
46-47 years old	2.34	1.40	3.88						
48-49 years old	2.25	1.33	3.80						

Note: 16953 individual observations used in the analysis.

Table S3: Ghana Demographic and Health Survey participants including those with missing BMI by survey year

	2003	2008	2014	2022
Women [N]	5256	4551	8717	13903
Age [Mean (SD)]	29.3 (9.81)	29.1 (9.87)	29.8 (9.91)	29.6 (9.83)
Birth year [Median (IQR)]	1975 (1965-1982)	1980 (1971-1988)	1985 (1976-1993)	1993 (1984-2001)

Note that in 2014 and 2022, only half of those surveyed were selected for biomarker collection.

Table S4: Sensitivity Analysis. Age-period-cohort regression results: Estimated odds (OR) of obesity compared to healthy weight and 95% confidence interval, Ghana (2003-2022) using 2-year age groups, single-year period and 7-year birth cohorts

Obesity	OR	95% Confidence Interval				OR	95% Confidence Interval		
2-YEAR AGE GROUP					*PERIOD (YEAR)*				
15-17 years old	0.36	0.22	0.59		2003	1	-	-	
18-19 years old	0.54	0.32	0.90		2008	1.46	1.16	1.84	
20-21 years old	1	-	-		2014	3.18	2.19	4.62	
22-23 years old	1.17	0.73	1.87		2022	5.21	2.86	9.49	
24-25 years old	1.65	1.03	2.64						
26-27 years old	3.03	1.91	4.80		*7-YEAR BIRTH COHORT*				
28-29 years old	2.94	1.77	4.87		1953-59	1	-	-	
30-31 years old	3.98	2.37	6.69		1960-66	0.82	0.56	1.19	
32-33 years old	4.60	2.71	7.79		1967-73	0.67	0.40	1.13	
34-35 years old	4.52	2.45	8.37		1974-80	0.55	0.28	1.08	
36-37 years old	4.95	2.54	9.64		1981-87	0.46	0.19	1.10	
38-39 years old	6.24	3.15	12.36		1988-94	0.41	0.14	1.22	
40-41 years old	5.83	2.89	11.76		1995-01	0.35	0.10	1.27	
42-43 years old	5.51	2.40	12.65		2001-07	0.37	0.08	1.64	
44-45 years old	4.10	1.73	9.71						
46-47 years old	4.69	1.96	11.25						
48-49 years old	3.94	1.50	10.37						

Table S5: Sensitivity Analysis. Age-period-cohort regression results: Estimated odds (OR) of overweight compared to healthy weight and 95% confidence interval, Ghana (2003-2022) using 2-year age groups, single-year period and 7-year birth cohorts

Obesity	OR	95% Confidence Interval				OR	95% Confidence Interval		
2-YEAR AGE GROUP					*PERIOD (YEAR)*				
15-17 years old	0.46	0.35	0.61		2003	1	-	-	
18-19 years old	0.79	0.60	1.04		2008	1.24	1.05	1.46	
20-21 years old	1	-	-		2014	1.62	1.22	2.16	
22-23 years old	1.35	1.04	1.75		2022	1.68	1.07	2.65	
24-25 years old	1.86	1.43	2.40						
26-27 years old	2.02	1.54	2.64		*7-YEAR BIRTH COHORT*				
28-29 years old	2.78	2.01	3.85		1953-59	1	-	-	
30-31 years old	2.66	1.88	3.77		1960-66	1.07	0.78	1.47	
32-33 years old	3.28	2.31	4.65		1967-73	1.08	0.71	1.63	
34-35 years old	3.04	2.00	4.63		1974-80	1.26	0.74	2.15	
36-37 years old	3.44	2.16	5.48		1981-87	1.38	0.70	2.73	
38-39 years old	3.42	2.10	5.55		1988-94	1.40	0.61	3.19	
40-41 years old	3.60	2.19	5.91		1995-01	1.32	0.49	3.53	
42-43 years old	3.50	1.92	6.36		2001-07	1.32	0.42	4.14	
44-45 years old	3.50	1.87	6.54						
46-47 years old	3.43	1.81	6.50						
48-49 years old	3.43	1.70	6.93						

Table S6: Sensitivity Analysis. Age-period-cohort regression results: Estimated odds (OR) of obesity compared to healthy weight and 95% confidence interval, Ghana (2003-2022) using 5-year age groups, single year period and 10-year birth cohorts

Obesity	OR	95% Confidence Interval				OR	95% Confidence Interval		
5-YEAR AGE GROUP					*PERIOD (YEAR)*				
15-19 years old	0.35	0.25	0.50		2003	1	-	-	
20-24 years old	1	-	-		2008	1.42	1.15	1.74	
25-29 years old	2.77	2.11	3.63		2014	2.95	2.25	3.87	
30-34 years old	4.52	3.34	6.13		2022	4.63	3.13	6.84	
35-39 years old	5.46	3.72	8.02						
40-45 years old	6.03	3.90	9.34		*10-YEAR BIRTH COHORT*				
46-49 years old	4.45	2.58	7.65		1953-59	1	-	-	
					1960-69	0.81	0.57	1.15	
					1970-79	0.68	0.43	1.09	
					1980-89	0.53	0.29	0.97	
					1990-99	0.45	0.21	0.99	
					2000-07	0.60	0.23	1.57	

Table S7: Sensitivity Analysis. Age-period-cohort regression results: Estimated odds (OR) of overweight compared to healthy weight and 95% confidence interval, Ghana (2003-2022) using 5-year age groups, single year period and 10-year birth cohorts

Obesity	OR	95% Confidence Interval				OR	95% Confidence Interval		
5-YEAR AGE GROUP					*PERIOD (YEAR)*				
15-19 years old	0.51	0.42	0.61		2003	1	-	-	
20-24 years old	1	-	-		2008	1.42	1.23	1.64	
25-29 years old	1.55	1.31	1.84		2014	2.15	1.76	2.63	
30-34 years old	1.85	1.50	2.27		2022	2.78	2.07	3.73	
35-39 years old	1.69	1.28	2.23						
40-45 years old	1.68	1.22	2.33		*10-YEAR BIRTH COHORT*				
46-49 years old	1.40	0.93	2.10		1953-59	1	-	-	
					1960-69	0.90	0.67	1.21	
					1970-79	0.75	0.52	1.10	
					1980-89	0.67	0.41	1.08	
					1990-99	0.52	0.28	0.95	
					2000-07	0.38	0.18	0.79	

Figure S1: Sensitivity Analysis. Age-period-cohort results using 2-year age groups, single-year period and 7-year birth cohorts

Obesity (BMI > 30) vs *Healthy weight (BMI ≤ 25)*		
a. Age	b. Period	c. Birth cohort
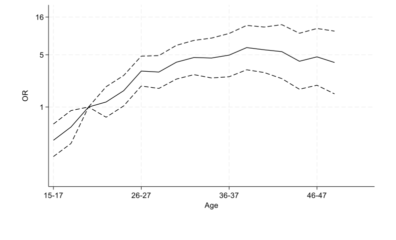	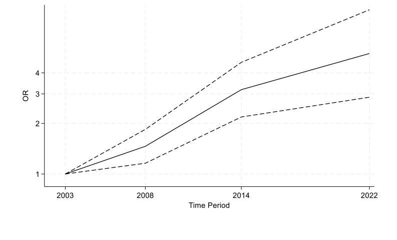	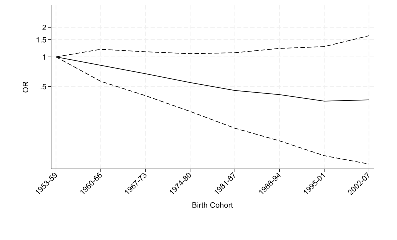
Overweight (25 < BMI ≤ 30) vs *Healthy weight (BMI ≤ 25)*		
a. Age	b. Period	c. Birth cohort
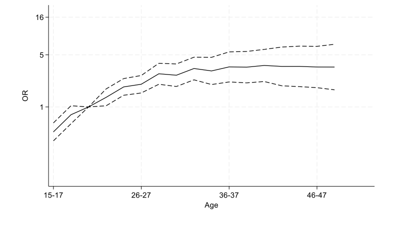	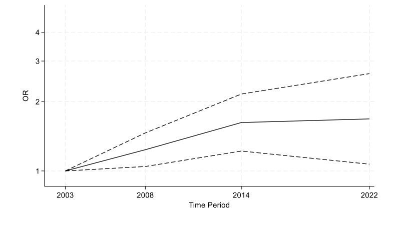	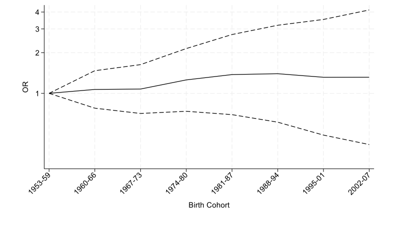

Odds Ratios (OR) – solid line, 95% confidence intervals - dashed lines. (a) age effects, (b) period effects, (c) cohort effects for the APC analysis for obesity (BMI > 30) vs Healthy weight (18.5 < BMI ≤ 25) (n=14998), (d) age effects, (e) period effects, (f) cohort effects for the APC analysis for overweight (25 < BMI ≤ 30) vs Healthy weight (18.5 < BMI ≤ 25) (n=16953).

Figure S2: Sensitivity Analysis. Age-period-cohort results using 5-year age groups, single year period and 10-year birth cohorts

Obesity (BMI > 30) vs *Healthy weight (BMI ≤ 25)*

a. Age

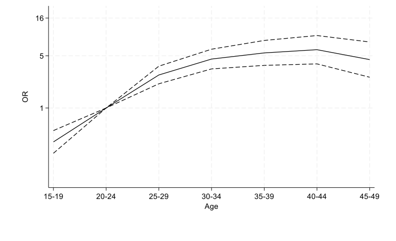

b. Period

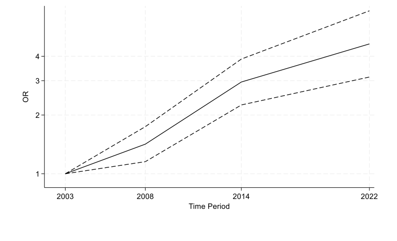

c. Birth cohort

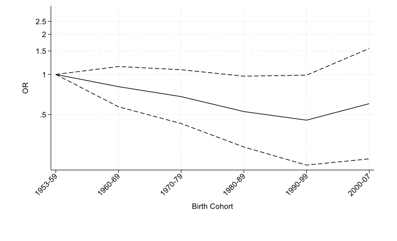

Overweight (25 < BMI ≤ 30) vs *Healthy weight (BMI ≤ 25)*

a. Age

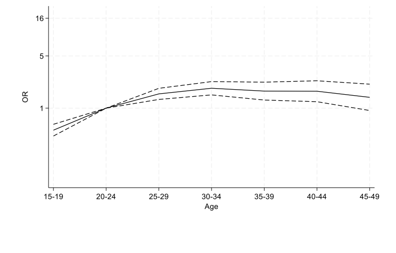

b. Period

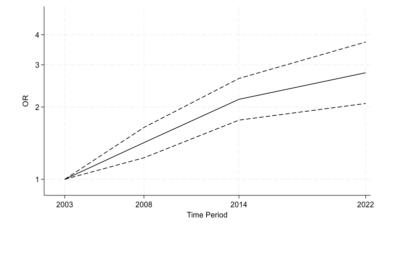

c. Birth cohort

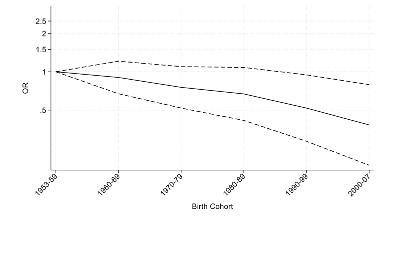

Odds Ratios (OR) – solid line, 95% confidence intervals - dashed lines. (a) age effects, (b) period effects, (c) cohort effects for the APC analysis for obesity (BMI > 30) vs Healthy weight (18.5 < BMI ≤ 25) (n=14998), (d) age effects, (e) period effects, (f) cohort effects for the APC analysis for overweight (25 < BMI ≤ 30) vs Healthy weight (18.5 < BMI ≤ 25) (n=16953).

Table S8: Underweight vs Healthy Weight. Age-period-cohort regression results: Estimated odds (OR) of underweight compared to healthy weight and 95% confidence interval, Ghana (2003-2022)

Obesity	OR	95% Confidence Interval				OR	95% Confidence Interval		
2-YEAR AGE GROUP					*PERIOD (YEAR)*				
15-17 years old	2.70	2.10	3.48		2003	1	-	-	
18-19 years old	1.51	1.14	2.00		2008	1.13	0.91	1.41	
20-21 years old	1	-	-		2014	1.02	0.75	1.40	
22-23 years old	0.81	0.58	1.13		2022	1.50	0.92	2.45	
24-25 years old	1.02	0.70	1.47						
26-27 years old	0.87	0.57	1.31		*10-YEAR BIRTH COHORT*				
28-29 years old	0.83	0.54	1.26		1953-59	1	-	-	
30-31 years old	0.74	0.47	1.18		1960-69	0.67	0.43	1.05	
32-33 years old	0.64	0.40	1.05		1970-79	0.43	0.23	0.79	
34-35 years old	0.69	0.38	1.24		1980-89	0.30	0.14	0.67	
36-37 years old	0.72	0.39	1.31		1990-99	0.27	0.10	0.72	
38-39 years old	0.60	0.33	1.09		2000-07	0.25	0.07	0.84	
40-41 years old	0.74	0.38	1.43						
42-43 years old	0.69	0.36	1.33						
44-45 years old	0.48	0.22	1.04						
46-47 years old	0.45	0.20	1.00						
48-49 years old	0.50	0.22	1.15						

Figure S3: Underweight vs Healthy Weight. Age-period-cohort results using HSE age groups, single year period and 10-year birth cohorts

Underweight (BMI ≤ 18.5) vs *Healthy weight (18.5 < BMI ≤ 25)*

a. Age	b. Period	c. Birth cohort

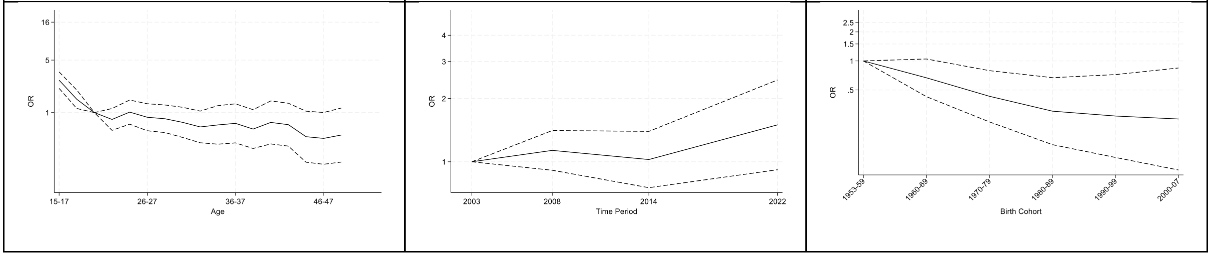

Odds Ratios (OR) – solid line, 95% confidence intervals - dashed lines. (a) age effects, (b) period effects, (c) cohort effects for the APC analysis for Underweight (BMI ≤ 18.5) vs Healthy weight (18.5 < BMI ≤ 25) (n=14272)
