# Supplementary material for: Long-term trends in obesity and overweight in women in Ghana from 2003–2023
Source: Commun Med (Lond). 2025 Aug 7;5:338. doi: 10.1038/s43856-025-01082-4 (PMC12332120; doi:10.1038/s43856-025-01082-4)
Supplement: Supplementary file 3 — Description of Additional Supplementary files [file 43856_2025_1082_MOESM3_ESM.pdf]

Description of Additional Supplementary files

File name: Supplementary Data 1

Description: Contains the source data for Figure 3.
