# Supplementary material for: Long-term trends in obesity and overweight in women in Ghana from 2003–2023
Source: Commun Med (Lond). 2025 Aug 7;5:338. doi: 10.1038/s43856-025-01082-4 (PMC12332120; doi:10.1038/s43856-025-01082-4)
Supplement: Supplementary file 5 — Reporting Summary [file 43856_2025_1082_MOESM5_ESM.pdf]

nature portfolio

Corresponding author(s): Laura A Gray

Last updated by author(s): Jul 30, 2025

Reporting Summary

Nature Portfolio wishes to improve the reproducibility of the work that we publish. This form provides structure for consistency and transparency in reporting. For further information on Nature Portfolio policies, see our Editorial Policies and the Editorial Policy Checklist.

Statistics

For all statistical analyses, confirm that the following items are present in the figure legend, table legend, main text, or Methods section.

n/a	Confirmed	
☐	☒	The exact sample size (*n*) for each experimental group/condition, given as a discrete number and unit of measurement
☐	☒	A statement on whether measurements were taken from distinct samples or whether the same sample was measured repeatedly
☐	☒	The statistical test(s) used AND whether they are one- or two-sided<br>*Only common tests should be described solely by name; describe more complex techniques in the Methods section.*
☐	☒	A description of all covariates tested
☐	☒	A description of any assumptions or corrections, such as tests of normality and adjustment for multiple comparisons
☐	☒	A full description of the statistical parameters including central tendency (e.g. means) or other basic estimates (e.g. regression coefficient) AND variation (e.g. standard deviation) or associated estimates of uncertainty (e.g. confidence intervals)
☒	☐	For null hypothesis testing, the test statistic (e.g. *F*, *t*, *r*) with confidence intervals, effect sizes, degrees of freedom and *P* value noted<br>*Give P values as exact values whenever suitable.*
☒	☐	For Bayesian analysis, information on the choice of priors and Markov chain Monte Carlo settings
☒	☐	For hierarchical and complex designs, identification of the appropriate level for tests and full reporting of outcomes
☒	☐	Estimates of effect sizes (e.g. Cohen's *d*, Pearson's *r*), indicating how they were calculated

Our web collection on statistics for biologists contains articles on many of the points above.

Software and code

Policy information about availability of computer code

Data collection	The data used are publicly available and were obtained from the Demographic and Health Surveys (DHS) Programme. Access to the data required registration and approval at http://dhsprogram.com/data/.
Data analysis	Data were analysed using Stata v18. Code used to analyse the data can be made available on request.

For manuscripts utilizing custom algorithms or software that are central to the research but not yet described in published literature, software must be made available to editors and reviewers. We strongly encourage code deposition in a community repository (e.g. GitHub). See the Nature Portfolio guidelines for submitting code & software for further information.

Data

Policy information about availability of data

All manuscripts must include a data availability statement. This statement should provide the following information, where applicable:

- Accession codes, unique identifiers, or web links for publicly available datasets
- A description of any restrictions on data availability
- For clinical datasets or third party data, please ensure that the statement adheres to our policy

The data used are publicly available and were obtained from the Demographic and Health Surveys (DHS) Programme. Access to the data required registration and approval at http://dhsprogram.com/data/.

Human research participants

Policy information about studies involving human research participants and Sex and Gender in Research.

Reporting on sex and gender	This study focuses on females (sex) only.
Population characteristics	See Above
Recruitment	Participants were recruited by the Ghana Statistical Services with technical support from the DHS Program. Harder to reach communities may be under-represented. Weights are available in the data but have previously been shown to make little difference to analysis.
Ethics oversight	The GSS submitted the survey protocol to the Ethical Review Committee (ERC) of the Ghana Health Service to ensure that the survey procedures were in accordance with Ghana's ethical research standards. The ERC granted ethical clearance for the survey.<br><br>ICF submitted the GDHS survey protocol to the ICF Institutional Review Board (IRB) to obtain ethical clearance and ensure that the survey procedures were in accordance with U.S. and international ethical research standards. The IRB provided ethical clearance for the survey.

Note that full information on the approval of the study protocol must also be provided in the manuscript.

Field-specific reporting

Please select the one below that is the best fit for your research. If you are not sure, read the appropriate sections before making your selection.

☐ Life sciences ☒ Behavioural & social sciences ☐ Ecological, evolutionary & environmental sciences

For a reference copy of the document with all sections, see nature.com/documents/nr-reporting-summary-flat.pdf

Behavioural & social sciences study design

All studies must disclose on these points even when the disclosure is negative.

Study description	Quantitative Age-Period-Cohort Analysis
Research sample	Women in Ghana between 2003 and 2022, ages between 15 and 49 years (reproductive age). Data taken from the Ghana Demographic and Health Survey.
Sampling strategy	Pregnant women were excluded since BMI is invalid during pregnancy. The sample was restricted to women between 15 and 49 years as this is considered reproductive age.
Data collection	Weight measurements were taken using SECA 874U scales with a digital display, and height was measured using a ShorrBoard® measuring board. The height of each participant was recorded by a measurer and assistant pair, and recorded on a form.
Timing	Cross sectional data collected in 2003, 2008, 2014 and 2022
Data exclusions	2590 were removed due to being pregnant because BMI is not a valid measure during pregnancy. 1579 were removed due to being underweight. This study looks at obesity and uses a comparison of healthy weight and so underweight individuals are considered to have different health problems. 11590 were removed due to having invalid BMI measurements.
Non-participation	The response rate for the data collected in 2003, 2008, 2014 and 2022 was 95.7%, 96.5%, 97.3%, and 98.0% respectively.
Randomization	Participants were not allocated into experimental groups.

Reporting for specific materials, systems and methods

We require information from authors about some types of materials, experimental systems and methods used in many studies. Here, indicate whether each material, system or method listed is relevant to your study. If you are not sure if a list item applies to your research, read the appropriate section before selecting a response.

Materials & experimental systems

n/a	Involved in the study
☒	☐ Antibodies
☒	☐ Eukaryotic cell lines
☒	☐ Palaeontology and archaeology
☒	☐ Animals and other organisms
☒	☐ Clinical data
☒	☐ Dual use research of concern

Methods

n/a	Involved in the study
☒	☐ ChIP-seq
☒	☐ Flow cytometry
☒	☐ MRI-based neuroimaging
